# Supplementary material for: Intellectual enrichment and genetic modifiers of cognition and brain volume in Huntington’s disease
Source: Brain Commun. 2022 Oct 31;4(6):fcac279. doi: 10.1093/braincomms/fcac279 (PMC9732861; doi:10.1093/braincomms/fcac279)
Supplement: fcac279_Supplementary_Data [file fcac279_supplementary_data.pdf]

Supplementary Methods

Occupational cognitive requirements

As a measure of occupational intellectual activity we used the occupational cognitive requirements of an individual's main occupation. We used the approach developed by Pool and colleagues[1] using O*NET scores, which was shown to predict cognitive function in healthy older adults. Primary occupation at the Visit of assessment or main occupation, if an individual had retired, was matched to O*NET-SOC occupation titles (version 23.2[2]). We then created a measure of occupational requirements by averaging the scores from the 10 cognitive related O*NET items: processing information, thinking creatively, judging the qualities of things, services or people, evaluating information to determine compliance with standards, analyzing data or information, making decisions and solving problems, updating and using relevant knowledge, developing objectives and strategies, scheduling work and activities, organizing, planning and prioritizing. Higher scores indicate higher cognitive requirements. Non-paid occupations such as housewife or students are not included in O*NET and were not given a score. They were 3 such cases in our cohort. In 7 cases it was not possible to find a clear match with O*NET occupation titles, because the occupation recorded was unclear, e.g. financial expert. These cases were therefore excluded for the analyses.

Control risk factors

The use of antipsychotic mediation has been associated with cognitive impairment[3]. In our cohort, there were 34 (14.8%) participants on antipsychotic medication on their baseline visit. The antipsychotic medication they were prescribed were: olanzapine, risperidone, aripiprazole, quetiapine, sulpiride, tiapride, amisulpride, tetrabenazine, paliperidone, clozapine and pimozide. The use of antipsychotic medication had a negative effect on cognitive function in our cohort ($p < 0.001$; parameter estimate (95% CI) = -0.855 (-1.30, -0.41); supplementary table 18). We therefore included it as a control variable in all the models with cognitive function as an outcome measure.

The presence of depression has also been associated with impaired cognitive function[4]. There were 28 (12.6%) participants who scored greater than 7 on the HADS[5], indicating the presence of depression, whereas for 6 participants there was no data on the HADS. The presence of depression was not a significant predictor of cognitive function in our cohort ($p > 0.05$; see supplementary table 18).

Lastly, we examined ApoE to test for concurrent risk of Alzheimer's dementia (AD) in e4 allele carriers in our cohort[6]. To determine APoE alleles in the Track-HD cohort rs429358 and rs7412 were imputed in Minimac4 using HRC.r1-1.GRCh37. A binary variable was created contrasting carriers and non-carriers of the detrimental e4 allele. There were 5 ambiguous cases in our cohort, which were either e2/e4 or e1/e3, and 16 participants with missing data. These were excluded from our analyses. In total, there were 60 (28.8%) e4 allele carriers in our cohort. Analyses testing for the effect of carrying the e4 allele on cognitive function in our cohort showed that it was not a significant predictor ($p > 0.09$; supplementary Table 18) and was therefore not included in any analyses.

Supplementary Tables

Supplementary Table 1: Control group demographics

**Characteristic**	
Total number at baseline	123
Male, No (%)	55 (45%)
Age at baseline, mean (SD)	46.1 (10.3)
Education Level, median (min - max)	4 (1, 6)
Verbal IQ, median (min – max)	38 (9 – 50)
SDMT at baseline median (min – max)	54 (30– 78)

Supplementary Table 2: Coding of binary genetic predictors

Variant	Gene being tagged	Homozygote minor allele and predictor code	Heterozygote and predictor code	Homozygote major allele and predictor code
rs2140734	*FAN1*	GG: 1	TG: 1	TT: 0
	MSH3	3a3a: 1	3aXa: 1	XaXa:0
rs9468	*MAPT*	CC (H2H2): 1	CT (H2H1): 1	TT (H1H1): 0
rs4680	*COMT*	AA (Met158Met): 1	AG (Val158Met): 0	GG (Val158Val): 0
rs6265	*BDNF*	TT (Met66Met): 1	TC (Met66Val): 1	CC (Val66Val): 0

Table showing how the different genetic predictors coded the different combination of alleles.
Xa: stands for 6a, 7a or 8a alleles.

Supplementary Table 3: Regression coefficients for intellectual enrichment and DBS model with cognitive function as outcome variable

**Parameter**	**Estimate**	**SE**	**DF**	**T-value**	**P-value**
Intercept	-4.19400	0.37410	220.9	-11.212	<0.001
Visit	0.23080	0.12320	604.9	1.873	0.061
$Visit^2$	-0.14450	0.03317	425.0	-4.357	<0.001
DBS	-0.02394	0.00246	232.8	-9.746	<0.001
Age	-0.13680	0.01735	221.4	-7.889	<0.001
Site - London	1.33400	0.48640	217.2	2.742	0.007
Site - Paris	0.19150	0.49300	221.7	0.388	0.698
Site - Vancouver	0.59340	0.48070	217.6	1.234	0.218
Sex - Male	-0.58060	0.35320	217.4	-1.644	0.102
Antipsychotic medication	-0.84600	0.21840	742.8	-3.874	<0.001
Intellectual Enrichment	0.52510	0.07620	217.7	6.890	<0.001
Intellectual Enrichment by DBS	-0.00207	0.00101	217.9	-2.052	0.041
Visit by Intellectual Enrichment	-0.01167	0.01713	213.4	-0.681	0.497
Visit by Intellectual Enrichment by DBS	-0.00083	0.00023	225.1	-3.596	<0.001
Visit by Age	-0.00588	0.00376	201.8	-1.562	0.120
Visit by DBS	-0.00802	0.00143	525.8	-5.626	<0.001
Visit by Site - London	-0.19400	0.10720	205.7	-1.810	0.072
Visit by Site – Paris	-0.01218	0.10760	203.7	-0.113	0.910
Visit by Site - Vancouver	-0.21800	0.10620	212.0	-2.053	0.041
$Visit^2$ by DBS	0.00141	0.00045	429.9	3.119	0.002

Supplementary Table 4: Regression coefficients for model with intellectual enrichment and group terms, with cognitive function as outcome variable

**Parameter**	**Estimate**	**SE**	**DF**	**T-value**	**P-value**
Intercept	-3.947837	0.353470	222.5	-11.169	<0.001
Visit	0.307414	0.125318	602.5	2.453	<0.001
Visit^2	-0.152926	0.033323	427.9	-4.589	<0.001
Group	-3.009590	0.269153	234.1	-11.182	<0.001
Age	-0.052558	0.018763	219.1	-2.801	0.006
Site - London	1.466529	0.466580	218.4	3.143	0.002
Site - Paris	0.430566	0.474181	222.7	0.908	0.365
Site - Vancouver	0.380672	0.459623	218.7	0.828	0.408
Sex - Male	-0.820579	0.338105	218.8	-2.427	0.016
Antipsychotic medication	-0.865641	0.218431	747.2	-4.589	<0.001
Intellectual Enrichment	0.507534	0.074440	218.7	6.818	<0.001
Intellectual Enrichment by Group	0.010694	0.103148	218.4	0.104	0.918
Visit by Intellectual Enrichment	-0.009345	0.018207	217.7	-0.513	0.608
Visit by Intellectual Enrichment by Group	-0.004113	0.025552	216.4	-0.161	0.872
Visit by Age	0.003780	0.004437	205.9	0.852	0.395
Visit by Group	-0.580852	0.152329	550.1	-3.813	<0.001
Visit by Site - London	-0.201426	0.112600	212.0	-1.789	0.075
Visit by Site – Paris	0.001570	0.113417	210.6	0.014	0.989
Visit by Site - Vancouver	-0.269283	0.110897	217.8	-2.428	0.016
Visit^2 by Group	0.067339	0.047191	428.4	1.427	0.154

Supplementary Table 5: Regression coefficients for model with intellectual enrichment and education terms, with cognitive function as outcome variable

Parameter	Estimate	SE	DF	T-value	P-value
Intercept	-4.278000	0.382500	220.7	-11.182	<0.001
Visit	0.249800	0.124800	599.1	4.780	<0.001
$Visit^2$	-0.144900	0.033160	425.4	-4.371	<0.001
DBS	-0.023170	0.002518	232.4	-9.201	<0.001
Age	-0.140000	0.017440	221.4	-8.027	<0.001
Site - London	1.297000	0.483900	217.1	2.679	0.008
Site - Paris	0.284600	0.503100	220.7	0.566	0.572
Site - Vancouver	0.638600	0.486600	217.3	1.312	0.191
Sex - Male	-0.580300	0.352800	217.4	-1.645	0.101
Antipsychotic medication	-0.839300	0.218400	743.5	-3.842	<0.001
Education	-2.32400	0.243600	217.5	-0.954	0.341
Education by DBS	0.004251	0.003508	217.3	1.212	0.227
Visit by Education	0.045450	0.054390	207.7	0.836	0.404
Visit by Education by DBS	0.000725	0.000773	207.1	0.937	0.350
Intellectual Enrichment	0.627000	0.131200	217.8	4.780	<0.001
Intellectual Enrichment by DBS	-0.003946	0.001834	216.9	-2.152	0.033
Visit by Intellectual Enrichment	-0.003155	0.029410	206.9	-1.073	0.285
Visit by Intellectual Enrichment by DBS	-0.001137	0.000404	205.2	-2.817	0.005
Visit by Age	-0.005607	0.003784	200.9	-1.482	0.140
Visit by DBS	-0.007879	0.001434	531.7	-5.494	<0.001
Visit by Site - London	-0.192400	0.107000	206.2	-1.797	0.074
Visit by Site – Paris	-0.030520	0.109700	201.7	-0.278	0.781
Visit by Site - Vancouver	-0.235000	0.107300	210.4	-2.191	0.030
$Visit^2$ by DBS	0.001397	0.000451	430.1	3.094	0.002

Table 6: Association between intellectual enrichment with cognitive function adjusted for motor and depressive symptoms

Model	Df	AIC	Chisq	P-value
**Intellectual Enrichment – adjusting for TMS**				
Null model	22	3065.2		
+ Intellectual Enrichment	23	3034.2	33.2	<0.001
+ Intellectual Enrichment by Visit	24	3034.2	1.9	0.164
+ Intellectual Enrichment by DBS	25	3031.7	4.5	0.034
+ Intellectual Enrichment by DBS by Visit	26	3022.6	11.1	<0.001
**Intellectual Enrichment – adjusting for HADS depression**				
Null model	22	3077.9		
+ Intellectual Enrichment	23	3038.9	41.0	<0.001
+ Intellectual Enrichment by Visit	24	3040.4	0.5	0.469
+ Intellectual Enrichment by DBS	25	3038.6	3.8	0.051
+ Intellectual Enrichment by DBS by Visit	26	3027.3	13.2	<0.001

The null model for intellectual enrichment included TMS at baseline (top model) or the presence of depression at baseline (bottom model), age at baseline, site (3 dummy variables) and DBS at baseline with their interaction with Visit, and main effects of sex and use of antipsychotic medication. A quadratic term was also included for Visit, as well as its interaction with DBS.

Supplementary Table 7: Association between intellectual enrichment and *MSH3* with brain volume

Model	Df	AIC	Chisq	P-value	P-value Bonferroni cor.
**Intellectual Enrichment**					
**Outcome measure: Caudate Volume**					
Null model	17	-4395.5			
+ Intellectual Enrichment	18	-4394.6	1.1	0.293	0.586
+ Intellectual Enrichment by Visit	19	-4392.6	0.0	0.975	1
+ Intellectual Enrichment by DBS	20	-4397.0	6.4	0.012	0.024
+ Intellectual Enrichment by DBS by Visit	21	-4395.8	0.8	0.357	0.714
**Outcome measure: Total GM volume**					
Null model	19	639.3			
+ Intellectual Enrichment	20	641.3	0.0	0.913	1
+ Intellectual Enrichment by Visit	21	643.2	0.0	0.907	1
+ Intellectual Enrichment by DBS	22	642.6	2.6	0.106	0.212
+ Intellectual Enrichment by DBS by Visit	23	640.8	3.8	0.051	0.102
**MSH3**					
**Outcome measure: Caudate Volume**					
Null model	17	-4613.6			
+ MSH3	18	-4614.0	2.4	0.119	0.239
+ MSH3 by Visit	19	-4616.2	4.2	0.041	0.082
+ MSH3 by DBS	20	-4614.4	0.2	0.641	1
+ MSH3 by DBS by Visit	21	-4612.4	0.0	0.940	1
**Outcome measure: Total GM volume**					
Null model	19	654.6			
+ MSH3	20	656.4	0.2	0.650	1
+ MSH3 by Visit	21	646.5	11.8	<0.001	0.001
+ MSH3 by DBS	22	648.1	0.4	0.520	1
+ MSH3 by DBS by Visit	23	646.5	3.6	0.058	0.117

The null model included age at baseline, DBS at baseline, site (3 dummy variables), with their interaction with Visit, and the main effect of sex. The total GM volume models also included a quadratic term for Visit, as well as its interaction with DBS. P-values were Bonferroni corrected for two independent comparisons.

Supplementary Table 8: Regression coefficients for intellectual enrichment and DBS model with caudate volume as outcome variable

**Parameter**	**Estimate**	**SE**	**DF**	**T-value**	**P-value**
Intercept	-0.01627	0.00970	205.1	-1.676	0.095
Visit	-0.01023	0.00050	193.9	-20.469	<0.001
DBS	-0.00070	0.00010	205.0	-10.848	<0.001
Age	-0.00281	0.00050	205.0	-6.083	<0.001
Site - London	0.02763	0.01260	205.0	2.189	0.030
Site - Paris	0.05614	0.01300	205.0	4.323	<0.001
Site - Vancouver	-0.00268	0.01270	205.0	-0.210	0.834
Sex - Male	-0.02350	0.00930	204.9	-2.559	0.012
Intellectual Enrichment	0.00208	0.00200	205.0	1.041	0.299
Intellectual Enrichment by DBS	-0.00007	0.00003	205.0	-2.552	0.011
Visit by Intellectual Enrichment	0.000003	0.00010	195.0	0.031	0.975
Visit by Age	0.00016	0.00003	190.0	6.254	<0.001
Visit by DBS	-0.00002	0.000003	187.0	-6.255	<0.001
Visit by Site - London	0.00111	0.00070	186.3	1.619	0.107
Visit by Site – Paris	0.00076	0.00070	189.0	1.059	0.291
Visit by Site - Vancouver	-0.00055	0.00070	192.2	-0.789	0.431

Supplementary Table 9: Regression coefficients for intellectual enrichment and group model with caudate volume as outcome variable

Parameter	Estimate	SE	DF	T-value	P-value
Intercept	0.04489	0.01089	205.6	4.123	<0.001
Visit	-0.00860	0.00056	188.0	-15.329	<0.001
Group	-0.10450	0.01057	205.0	-9.884	<0.001
Age	-0.00072	0.00054	205.1	-1.346	0.180
Site - London	0.03067	0.01304	205.0	2.353	0.020
Site - Paris	0.06290	0.01343	205.0	4.683	<0.001
Site - Vancouver	-0.01060	0.01309	205.1	-0.810	0.419
Sex - Male	-0.03177	0.00410	204.8	-3.357	<0.001
Intellectual Enrichment	0.00288	0.00314	206.5	0.916	0.360
Intellectual Enrichment by Group	-0.00160	0.00410	205.1	-0.389	0.698
Visit by Intellectual Enrichment	0.00008	0.00011	194.3	0.066	0.947
Visit by Age	0.00021	0.00003	187.4	7.337	<0.001
Visit by Group	-0.00296	0.00057	184.4	-5.192	<0.001
Visit by Site - London	0.00121	0.00071	186.3	1.703	0.090
Visit by Site – Paris	0.00091	0.00073	188.4	1.237	0.217
Visit by Site - Vancouver	-0.00088	0.00072	191.3	-1.231	0.220

Supplementary Table 10: VBM results for intellectual enrichment and *MSH3*

**Region with most cluster voxels**	**Cluster Peak MNI Coordinates**			**Cluster Peak z-value**	**No. of Voxels in Cluster**	**Cluster P-value FWE-corrected**
	x	***y***	***z***			
**Intellectual Enrichment by DBS: baseline**						
Left Superior Temporal Gyrus	-50	-36	16	4.42	739	0.027
Thalamus bilaterally	6	-8	4	4.04	1075	0.005
Right Putamen	22	16	-10	3.63	860	0.015
**Intellectual Enrichment by DBS: change**						
Right Precuneus	18	-62	41	4.63	5080	<0.001
Right Superior Temporal Gyrus	55	-29	9			
Right Postcentral Gyrus	37	-26	40			
Right Superior Parietal Lobe	24	-48	58			
***MSH3*: baseline**						
Right Middle Temporal Gyrus	56	-32	-3	4.75	2156	<0.001
Left Post-Central Gyrus	-44	-30	45	4.33	844	0.015
Right Post-Central Gyrus	57	-18	38	4.25	625	0.048
***MSH3*: change**						
Right Fusiform Gyrus	37	-55	-16	5.18	3700	<0.001
Left Fusiform Gyrus	-32	-55	-14	4.54	3011	<0.001
Left Inferior Frontal Gyrus	-44	27	13	4.51	1520	<0.001
Left Precuneus	-15	-62	37	4.44	1211	0.002
Right Inferior Frontal Gyrus	26	29	-11	4.04	981	0.005
Right Middle Frontal Gyrus	40	18	45	4.16	526	0.049
***MSH3* by DBS: change**						
Left supplementary motor area	-5	14	49	4.43	3897	<0.001
Left inferior frontal gyrus	-40	-26	13	4.39		
Right superior temporal gyrus	52	-10	-6	4.23	1386	0.001

Significant clusters were identified using a threshold $p < 0.001$ voxel-uncorrected; $p < 0.05$ family-wise error (FWE) cluster-corrected. Region labels based on the AAL atlas[7] using the WFU Pickatlas[8].

Supplementary Table 11: Regression coefficients for *MSH3* model with cognitive function as outcome variable

Parameter	Estimate	SE	DF	T-value	P-value
Intercept	-4.80200	0.04485	217.3	-10.707	<0.001
Visit	0.21170	0.13120	576.5	1.613	0.107
$Visit^2$	-0.14060	0.03371	419.8	-4.169	<0.001
DBS	-0.02637	0.00276	229.1	-9.546	<0.001
Age	-0.12280	0.01939	216.2	-6.333	<0.001
Site - London	1.49100	0.55050	213.6	2.708	0.007
Site - Paris	0.70900	0.55300	217.4	1.282	0.201
Site - Vancouver	0.75470	0.55430	213.8	1.362	0.175
Sex - Male	-0.23600	0.39550	214.0	-0.597	0.551
Antipsychotic medication	-1.07000	0.24260	714.5	-4.412	<0.001
MSH3 - 3a allele carriers	1.06500	0.39950	215.7	2.666	0.008
Visit by *MSH3*	-0.21030	0.07973	198.7	2.637	0.009
Visit by Age	-0.00725	0.00389	200.6	-1.865	0.064
Visit by DBS	-0.00747	0.00147	516.9	-5.095	<0.001
Visit by Site - London	-0.23530	0.11170	202.4	-2.107	0.036
Visit by Site – Paris	-0.07850	0.11060	199.9	-0.710	0.479
Visit by Site - Vancouver	-0.22820	0.11260	208.2	-2.028	0.044
$Visit^2$ by DBS	0.00144	0.00046	418.9	3.106	0.002

Supplementary Table 12: Regression coefficients for *MSH3, FAN1, MAPT* and *COMT* models coding for the number of alleles with cognitive function as outcome variable

Model	Df	AIC	Chisq	P-value	P-value Bonferroni cor.
**MSH3**					
Null model	20	3139.0			
+ MSH3	21	3135.3	5.7	0.017	0.084
+ MSH3 by Visit	22	3130.6	6.7	0.010	0.049
+ MSH3 by DBS	23	3131.7	0.9	0.337	1
+ MSH3 by DBS by Visit	24	3133.7	0.0	0.855	1
**FAN1**					
Null model	20	3109.3			
+ FAN1	21	3110.4	0.9	0.936	1
+ FAN1 by Visit	22	3112.2	0.2	0.203	1
+ FAN1 by DBS	23	3113.6	0.6	0.580	1
+ FAN1 by DBS by Visit	24	3110.7	4.9	0.027	0.135
**MAPT**					
Null model	20	3109.3			
+ MAPT	21	3108.8	2.5	0.114	0.568
+ MAPT by Visit	22	3110.8	0.0	0.926	1
+ MAPT by DBS	23	3109.1	3.7	0.054	0.269
+ MAPT by DBS by Visit	24	3111.1	0.0	0.881	1
**COMT**					
Null model	20	3109.3			
+ COMT	21	3111.2	0.1	0.789	1
+ COMT by Visit	22	3112.7	0.5	0.461	1
+ COMT by DBS	23	3113.6	1.1	0.299	1
+ COMT by DBS by Visit	24	3115.4	0.2	0.691	1

Supplementary Table 13: Regression coefficients for *MSH3* model with total GM volume as outcome variable

**Parameter**	**Estimate**	**SE**	**DF**	**T-value**	**P-value**
Intercept	0.68890	0.45220	214.4	1.524	0.129
Visit	-0.09751	0.02095	342.0	-4.654	<0.001
$Visit^2$	-0.03038	0.00385	368.3	-7.901	<0.001
DBS	-0.01442	0.00279	208.8	-5.165	<0.001
Age	-0.20430	0.02009	209.3	-10.169	<0.001
Site - London	-0.71020	0.56120	208.8	-1.266	0.207
Site - Paris	-0.69810	0.56040	209.3	-1.246	0.214
Site - Vancouver	-0.02344	0.57760	210.2	-0.041	0.968
Sex - Male	-1.57800	0.35560	206.8	-4.437	<0.001
MSH3 - 3a allele carriers	0.85440	0.41310	209.3	2.069	0.040
Visit by *MSH3*	0.05859	0.01681	196.1	3.486	<0.001
Visit by Age	-0.00452	0.00083	198.3	-5.455	<0.001
Visit by DBS	-0.00055	0.00019	535.9	-2.925	0.004
Visit by Site - London	0.00929	0.02256	195.3	-5.455	<0.001
Visit by Site – Paris	-0.01274	0.02275	199.8	-0.560	0.576
Visit by Site - Vancouver	0.00110	0.02390	205.4	0.046	0.963
$Visit^2$ by DBS	-0.00013	0.00005	365.9	-2.479	0.013

Supplementary Table 14: Regression coefficients for *BDNF* and intellectual enrichment model with cognitive function as outcome variable

**Parameter**	**Estimate**	**SE**	**DF**	**T-value**	**P-value**
Intercept	-4.14600	0.41450	204.8	-10.003	<0.001
Visit	0.31950	0.13200	549.9	2.421	0.016
Visit^2	-0.13430	0.03418	398.2	-3.930	<0.001
DBS	-0.02267	0.00259	216.8	-8.764	<0.001
Age	-0.12860	0.01844	205.3	-6.974	<0.001
Site - London	1.49100	0.49980	202.0	2.984	0.003
Site - Paris	0.38720	0.51440	205.8	0.753	0.452
Site - Vancouver	0.58970	0.50400	202.2	1.170	0.243
Sex - Male	-0.49460	0.36980	202.3	-1.337	0.183
Antipsychotic medication	-0.96160	0.23710	686.7	-4.056	<0.001
Intellectual Enrichment	0.36410	0.10160	202.2	3.583	<0.001
BDNF – Met66 allele carriers	-0.21460	0.37470	202.3	-0.573	0.568
Intellectual Enrichment by *BDNF*	0.44540	0.16140	203.5	2.760	0.006
Visit by age	-0.00492	0.00410	189.7	-1.201	0.231
Visit by Site - London	-0.23570	0.11290	192.0	-2.088	0.0381
Visit by Site – Paris	-0.27030	0.11450	189.0	-0.236	0.814
Visit by Site - Vancouver	-0.21070	0.11390	196.5	-1.850	0.065
Visit by DBS	-0.00767	0.00150	494.2	-5.098	<0.001
Visit^2 by DBS	0.00142	0.00047	397.9	2.998	0.003
Visit by Intellectual Enrichment	-0.01213	0.01836	199.9	-0.661	0.510
Visit by *BDNF*	-0.18340	0.08423	190.5	-2.177	0.031

Supplementary Table 15: Interaction between intellectual enrichment and *BDNF* on brain volume

Model	Df	AIC	Chisq	P-value	P-value Bonferroni cor.
**Caudate volume**					
Null model	21	-4335.9			
+ *BDNF* by Intellectual Enrichment	22	-4334.1	0.2	0.654	1
+ *BDNF* by Intellectual Enrichment by Visit	23	-4338.1	6.0	0.014	0.029
**Total GM volume**					
Null model	23	645.7			
+ *BDNF* by Intellectual Enrichment	24	646.9	0.8	0.376	0.751
+ *BDNF* by Intellectual Enrichment by Visit	25	637.0	11.9	<0.001	0.001

The null model included intellectual enrichment, *BDNF*, age at baseline, site (3 dummy variables), DBS at baseline with their interaction with Visit, and sex. For the model with total GM volume as outcome measure a quadratic term was also included for Visit, as well as its interaction with DBS. P-values were corrected for 2 independent comparisons using Bonferroni correction.

Supplementary Table 16: Regression coefficients for the *BDNF* and intellectual enrichment model with caudate volume as outcome variable

**Parameter**	**Estimate**	**SE**	**DF**	**T-value**	**P-value**
Intercept	-0.01151	0.01066	203.2	-1.079	0.2818
Visit	0.01023	0.00053	187.3	-19.405	<0.001
DBS	-0.00067	0.00007	203.0	-10.203	<0.001
Age	0.00272	0.00047	203.0	-5.749	<0.001
Site - London	0.02705	0.01290	203.0	2.097	0.037
Site - Paris	0.05544	0.01320	203.0	4.199	<0.001
Site - Vancouver	-0.00426	0.01301	203.0	-0.327	0.744
Sex - Male	-0.02385	0.00953	202.9	-2.502	0.013
Intellectual Enrichment	0.00143	0.00262	203.0	0.546	0.585
BDNF – Met66 allele carriers	-0.00541	0.00967	203.0	-0.560	0.5762
Intellectual Enrichment by *BDNF*	0.00247	0.00416	203.0	0.595	0.553
Visit by Age	0.00016	0.00003	188.2	6.395	<0.001
Visit by DBS	-0.00002	0.000004	186.2	-6.539	<0.001
Visit by Site - London	0.00123	0.00068	185.6	1.801	0.073
Visit by Site – Paris	0.00090	0.00071	187.8	1.272	0.205
Visit by Site - Vancouver	-0.00056	0.00069	191.0	-0.811	0.419
Visit by Intellectual Enrichment	-0.00021	0.00014	187.7	-1.518	0.131
Visit by *BDNF*	-0.00038	0.00052	191.8	-0.727	0.468
Visit by Intellectual Enrichment by *BDNF*	0.00057	0.00023	203.8	2.466	0.015

Supplementary Table 17: Regression coefficients for the *BDNF* and intellectual enrichment model with total GM volume as outcome variable

**Parameter**	**Estimate**	**SE**	**DF**	**T-value**	**P-value**
Intercept	1.12900	0.47320	207.1	2.387	0.018
Visit	-0.07176	0.02103	332.0	-3.412	0.001
Visit^2	-0.03101	0.00406	347.5	-7.645	<0.001
DBS	-0.01205	0.00298	197.2	-4.042	<0.001
Age	-0.21060	0.02171	198.2	-9.700	<0.001
Site - London	-0.69290	0.58020	197.1	-1.194	0.234
Site - Paris	-0.64750	0.59370	197.3	-1.091	0.277
Site - Vancouver	-0.10070	0.59790	198.8	-0.168	0.866
Sex - Male	-1.48000	0.37870	194.5	-3.909	<0.001
Intellectual Enrichment	-0.04635	0.11830	199.2	-0.392	0.696
BDNF – Met66 allele carriers	-0.26430	0.43790	198.3	-0.603	0.547
Intellectual Enrichment by *BDNF*	0.16480	0.18900	197.8	0.872	0.384
Visit by Age	-0.00460	0.00088	186.9	-5.204	<0.001
Visit by DBS	-0.00042	0.00020	503.9	-2.075	0.039
Visit by Site - London	0.01646	0.02304	183.8	0.714	0.476
Visit by Site – Paris	-0.00322	0.02381	187.2	-0.135	0.893
Visit by Site - Vancouver	-0.00675	0.02426	190.5	-0.278	0.781
Visit by Intellectual Enrichment	-0.01016	0.00459	178.6	-2.212	0.028261
Visit by *BDNF*	-0.01294	0.01752	187.0	-0.738	0.461
Visit by Intellectual Enrichment by *BDNF*	0.02680	0.00763	193.0	3.511	<0.001
Visit^2 by DBS	-0.00015	0.00006	347.7	-2.701	0.007253

Supplementary Table 18: Association between control risk factors and cognitive function

Model	Df	AIC	Chisq	P-value
**Antipsychotic medication**				
Null model	19	3328.3		
+ Antipsychotic medication	20	3316.2	14.1	<0.001
+ Antipsychotic medication by Visit	21	3316.6	1.6	0.200
**Depression**				
Null model	19	3242.3		
+ Depression	20	3240.6	3.8	0.052
+ Depression by Visit	21	3242.5	0.0	0.913
**ApoE e3 allele carriers**				
Null model	19	3046.3		
+ Intellectual Enrichment	20	3048.3	0.1	0.794
+ Intellectual Enrichment by Visit	21	3047.4	2.8	0.092

The null model included age at baseline, DBS at baseline, site (3 dummy variables) with their interaction with Visit, and sex. A quadratic term was also included for Visit, as well as its interaction with DBS.

References

1. Pool LR, Weuve J, Wilson RS, Bültmann U, Evans DA, Mendes de Leon CF. Occupational cognitive requirements and late-life cognitive aging. *Neurology*. 2016;86(15):1386-1392. doi:10.1212/WNL.0000000000002569

2. Peterson NG, Mumford MD, Borman WC, Jeanneret PR, Fleishman EA, eds. *An Occupational Information System for the 21st Century: The Development of O*NET*. American Psychological Association; 1999:xii, 336. doi:10.1037/10313-000

3. Vigen CLP, Mack WJ, Keefe RSE, et al. Cognitive Effects of Atypical Antipsychotic Medications in Patients With Alzheimer's Disease: Outcomes From CATIE-AD. *Am J Psychiatry*. 2011;168(8):831-839. doi:10.1176/appi.ajp.2011.08121844

4. Smith MM, Mills JA, Epping EA, Westervelt HJ, Paulsen JS. Depressive Symptom Severity is Related to Poorer Cognitive Performance in Prodromal Huntington Disease. *Neuropsychology*. 2012;26(5):664-669. doi:10.1037/a0029218

5. Zigmond AS, Snaith RP. The Hospital Anxiety and Depression Scale. *Acta Psychiatr Scand*. 1983;67(6):361-370. doi:10.1111/j.1600-0447.1983.tb09716.x

6. Harris SE, Deary IJ. The genetics of cognitive ability and cognitive ageing in healthy older people. *Trends Cogn Sci*. 2011;15(9):388-394. doi:10.1016/j.tics.2011.07.004

7. Tzourio-Mazoyer N, Landeau B, Papathanassiou D, et al. Automated Anatomical Labeling of Activations in SPM Using a Macroscopic Anatomical Parcellation of the MNI MRI Single-Subject Brain. *NeuroImage*. 2002;15(1):273-289. doi:10.1006/nimg.2001.0978

8. Maldjian JA, Laurienti PJ, Kraft RA, Burdette JH. An automated method for neuroanatomic and cytoarchitectonic atlas-based interrogation of fMRI data sets. *NeuroImage*. 2003;19(3):1233-1239. doi:10.1016/S1053-8119(03)00169-1

Supplementary Figures

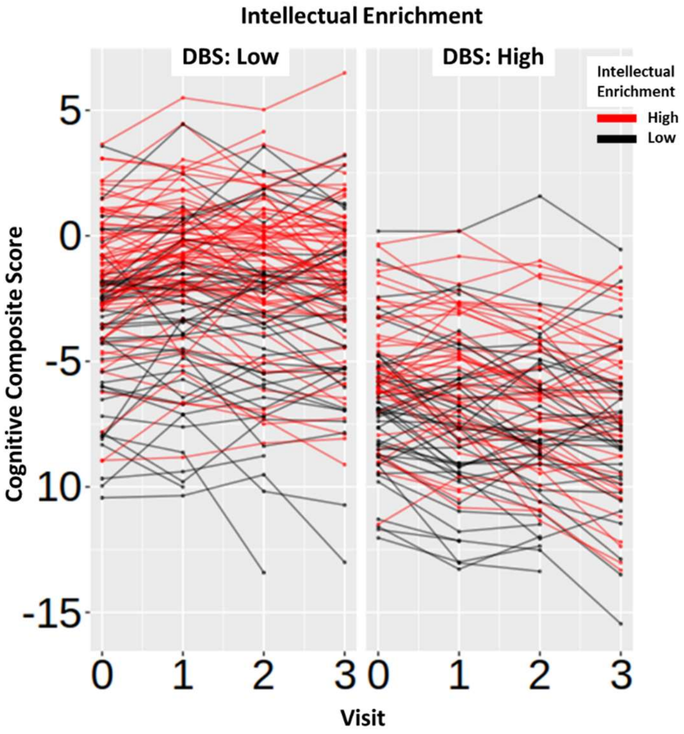

Supplementary Figure 1: Association between intellectual enrichment, DBS and global cognitive function. For visualization purposes results are split into high (above mean) and low (below mean) DBS. Individual lines are drawn for each participant and colour coded for high (above mean; red) and low (below mean; black) intellectual enrichment. Datapoints show the raw data residualized against age, site, sex and use of antipsychotic medication.

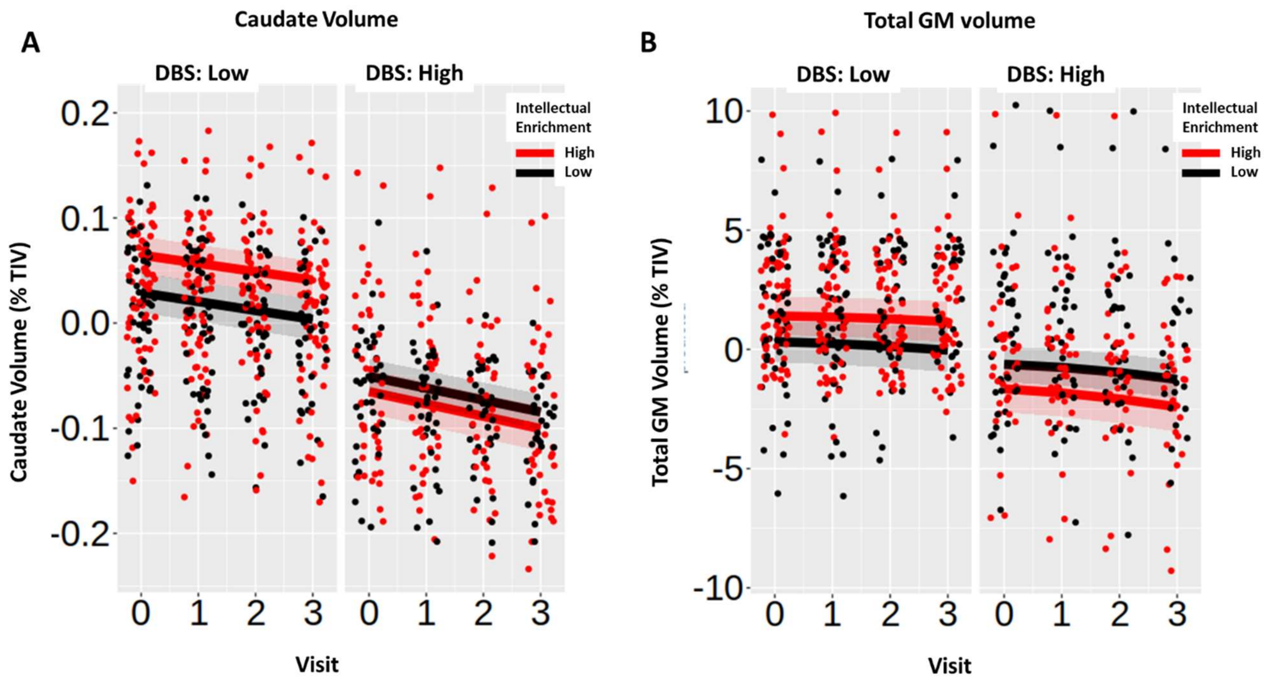

Supplementary Figure 2: (A) Association between intellectual enrichment and DBS with caudate volume (t-value(205.0) = -2.552, p-value = 0.011), and (B) total GM volume (p-value > 0.05) as percent of total intracranial volume (TIV). For visualization purposes results are split into high (above mean) and low (below mean) DBS. Regression lines are generated from the mixed linear model at high (1SD above mean; red) and low (1SD below mean; black) intellectual enrichment. Bands around the regression lines are 95% confidence intervals. Datapoints show the raw data residualized against age, site, sex and use of antipsychotic medication, and have been jittered to minimize overlap.

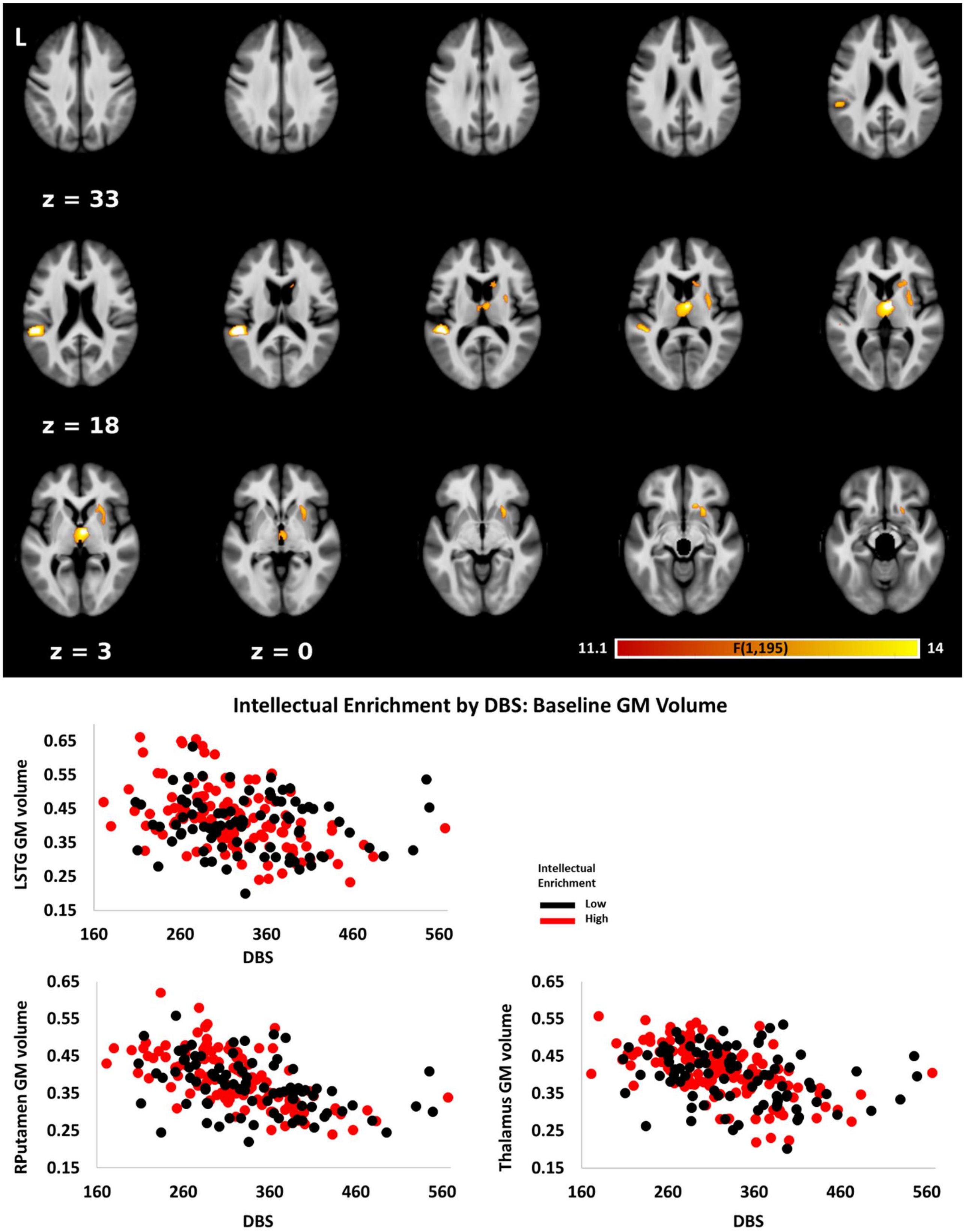
L
z = 33
z = 18
z = 3
z = 0
11.1
F(1,195)
14
Intellectual Enrichment by DBS: Baseline GM Volume
LSTG GM volume
DBS
Intellectual Enrichment
Low
High
RPutamen GM volume
DBS
Thalamus GM volume
DBS

Supplementary Figure 3: Association between intellectual enrichment and DBS with baseline GM volume. Shown on top are significant clusters overlaid on the group template. T-maps are thresholded at $p < 0.001$ uncorrected at voxel level and $p < 0.05$ family-wise error (FWE) corrected at cluster-level. Shown in a scatter plots (bottom) are the extracted values averaged across the 3 significant clusters. For visualization purposes data are grouped by high (above mean - red) and low (below mean - black) intellectually enrichment. Data have been adjusted for age, site, sex and TIV. LSTG = left superior temporal gyrus.

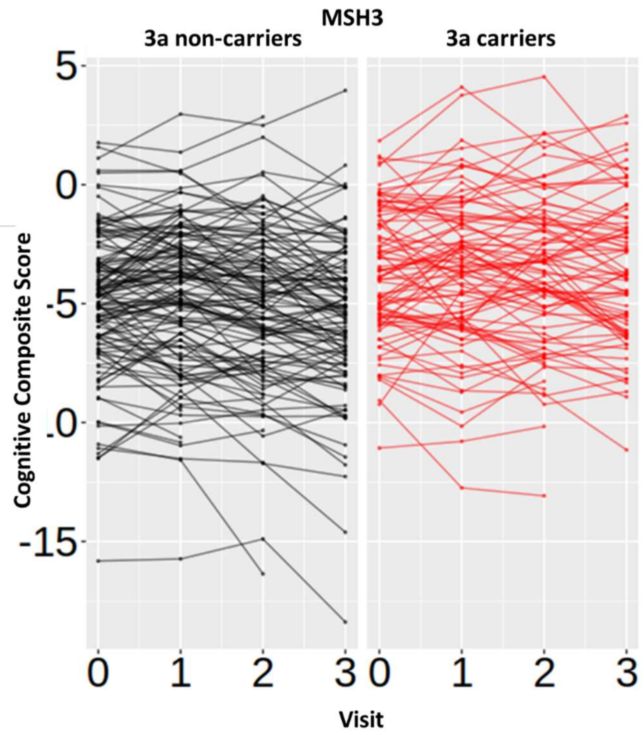

Supplementary Figure 4: Association between the MSH3 predictor with global cognitive function. For visualization purposes results are shown in separate panels for carriers (right, red) and non-carriers (left, black) of the 3a allele. Individual lines are drawn for each participant. Datapoints show the raw data residualized against age, DBS, site, sex and use of antipsychotic medication.

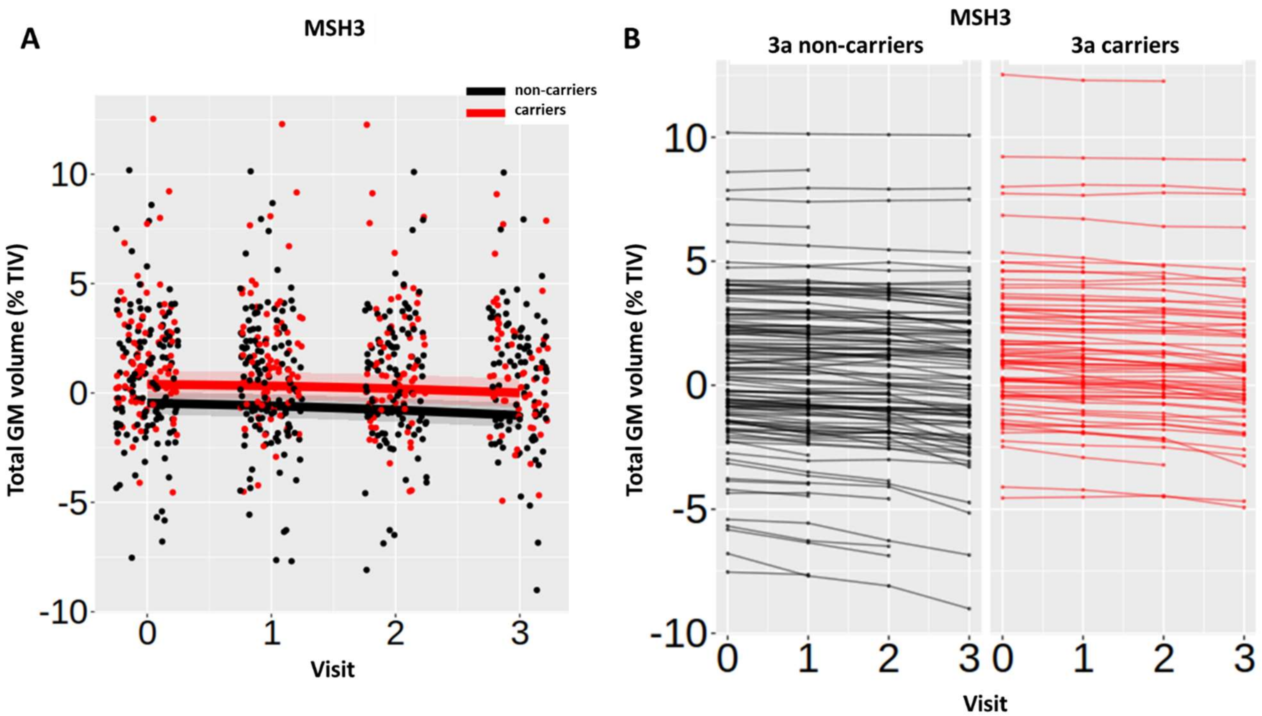

Supplementary Figure 5: Association between the MSH3 predictor with total GM volume as percent TIV (t-value(196.1) = 3.486, p-value < 0.001). In (A) regression lines are generated from the mixed linear model for carriers (red) and non-carriers (black) of the 3a allele. Bands around the regression lines are 95% confidence intervals. Datapoints have been jittered to minimize overlap. In (B) individual lines are drawn for each participant and colour coded for carriers (right, red) and non-carriers (left, black) of the 3a allele. Datapoints show the raw data residualized against age, DBS, site and sex.

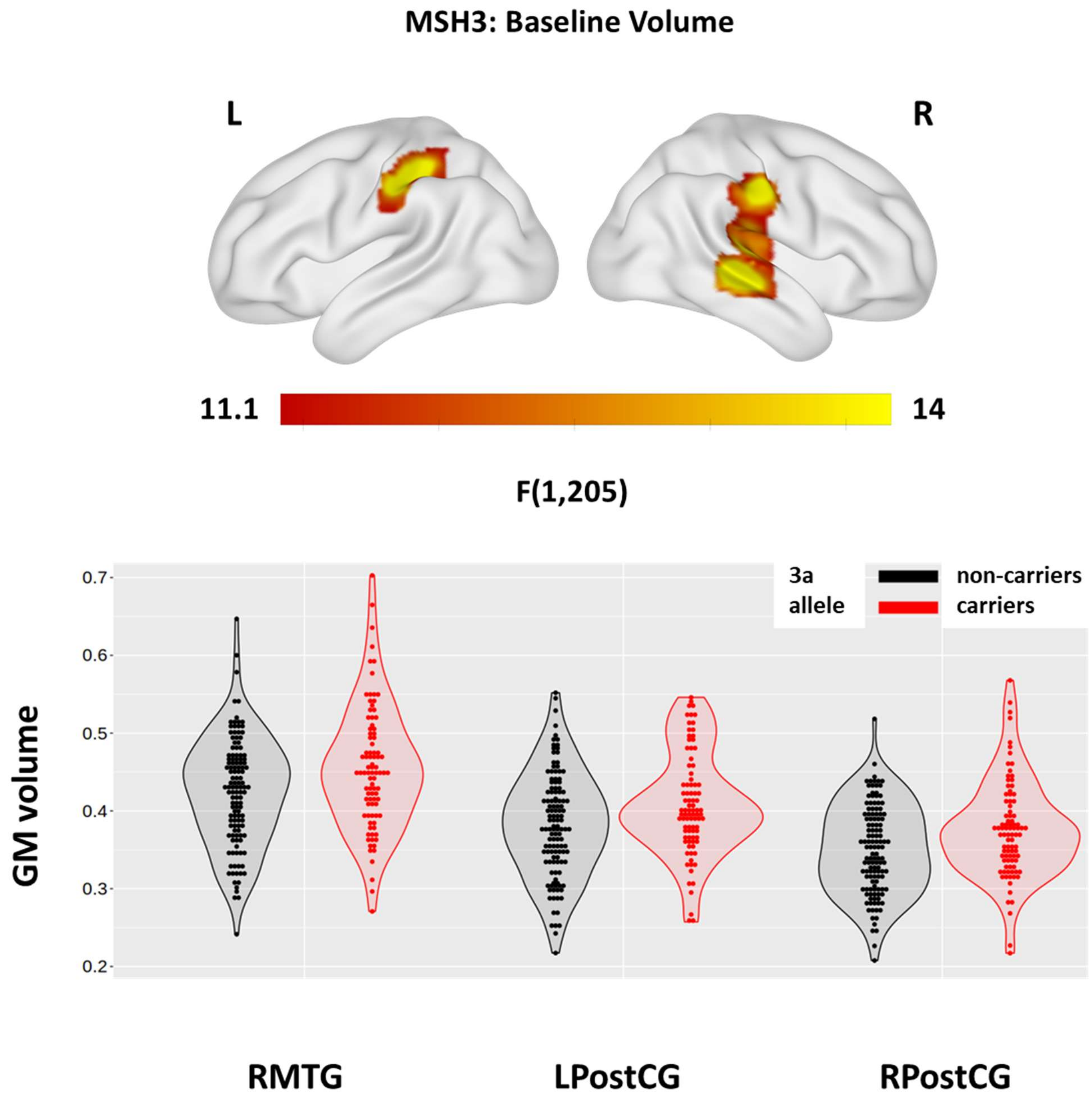

Supplementary Figure 6: Association between the *MSH3* predictor with baseline GM volume. Significant clusters are overlaid on the ICBM152 template mesh (top). T-maps are thresholded at $p < 0.001$ uncorrected at voxel level and $p < 0.05$ family-wise error (FWE) corrected at cluster-level. Shown in violin plots (bottom) are the extracted values averaged across the 3 significant clusters for carriers (red) and non-carriers (black) of the 3a allele. Individual datapoints are shown in black dots. Datapoints show the raw data residualized against age, DBS, site, sex and

TIV. RMTG = right middle temporal gyrus; LPostCG and RPostCG= left and right postcentral gyrus.

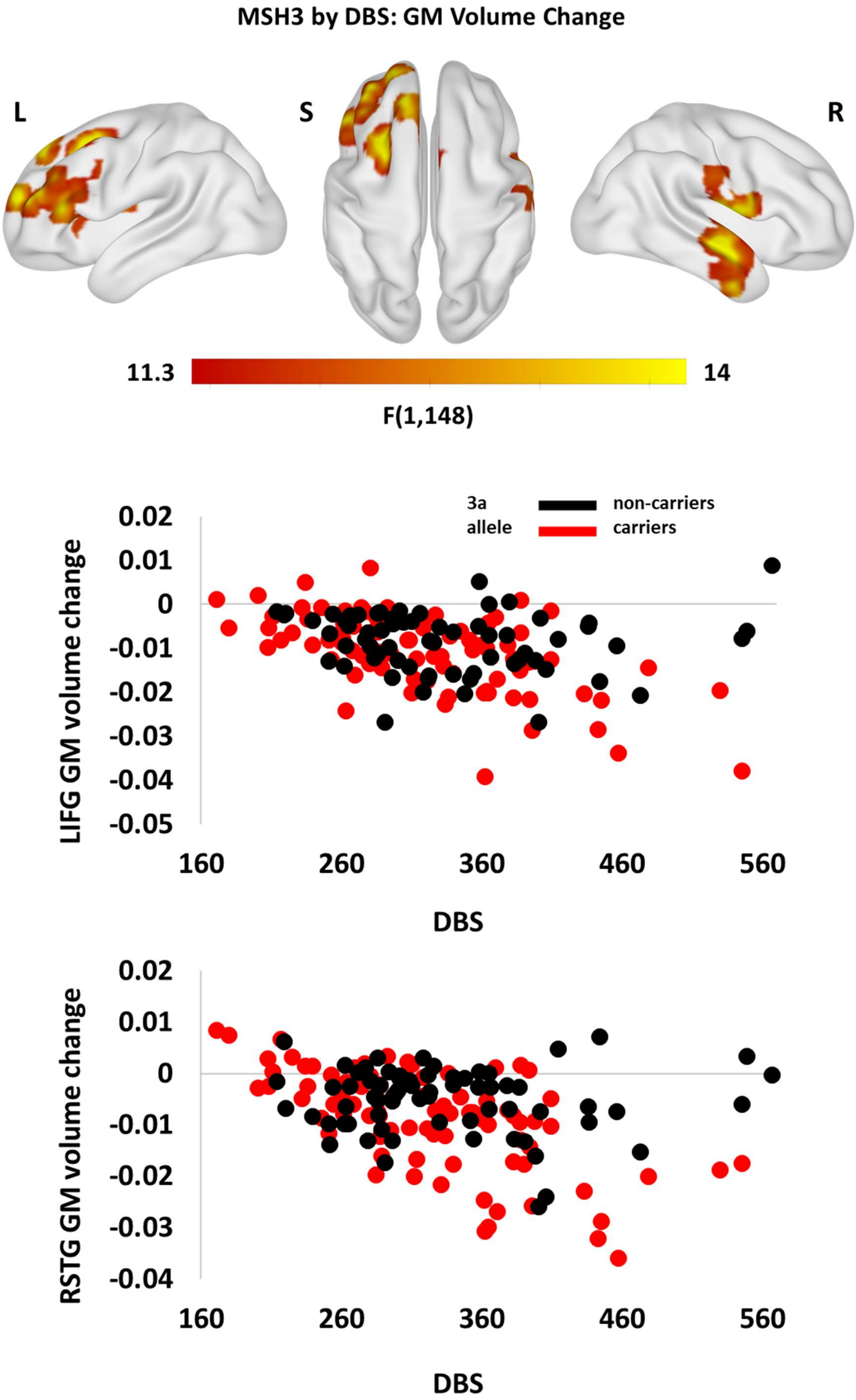
MSH3 by DBS: GM Volume Change
L
S
R
11.3
14
F(1,148)
3a
allele
non-carriers
carriers
LIFG GM volume change
0.02
0.01
0
-0.01
-0.02
-0.03
-0.04
-0.05
160
260
360
460
560
DBS
RSTG GM volume change
0.02
0.01
0
-0.01
-0.02
-0.03
-0.04
160
260
360
460
560
DBS

Supplementary Figure 7: Association between the *MSH3* predictor and DBS with 3-year GM volume change (B). Significant clusters are overlaid on the ICBM152 template mesh (top). T-maps are thresholded at $p < 0.001$ uncorrected at voxel level and $p < 0.05$ family-wise error (FWE) corrected at cluster-level. Shown in scatter plots (bottom) are the extracted values averaged across the significant clusters for carriers (red) and non-carriers (black) of the 3a allele. Datapoints show the raw data residualized against age, DBS, site, sex and TIV. RSTG = right superior temporal gyrus; LIFG = left and right inferior frontal gyrus.

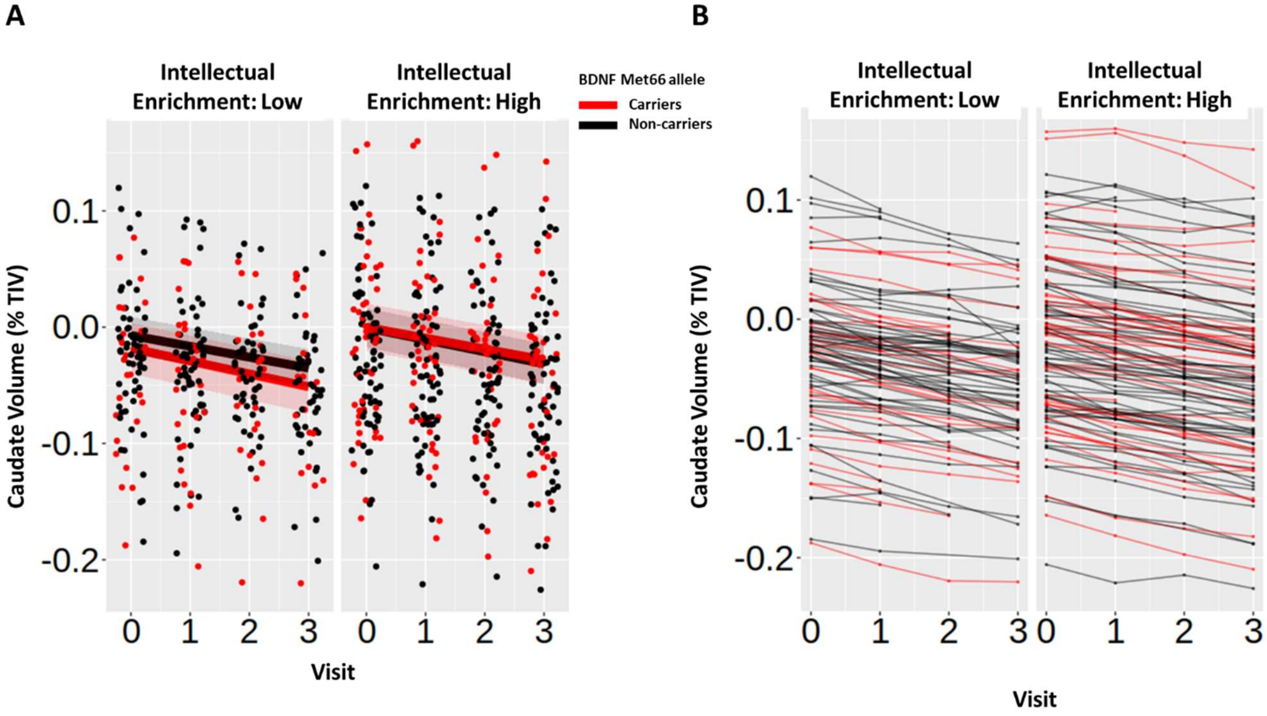

Supplementary Figure 8: Association between intellectual enrichment and *BDNF* with caudate volume as percent TIV (t-value(203.8) = 2.466, p-value = 0.015). For visualization purposes results are split into high (above mean) and low (below mean) intellectual enrichment. In (A) regression lines are generated from the mixed linear model for *BDNF* Met66 allele carriers (red) and non-carriers (black). Bands around the regression lines are 95% confidence intervals. Data have been jittered to minimize overlap. In (B) individual lines are drawn for each participant and colour coded for carriers (red) and non-carriers (black) of the Met66 allele. Datapoints in both plots show the raw data residualized against age, DBS, site and sex.

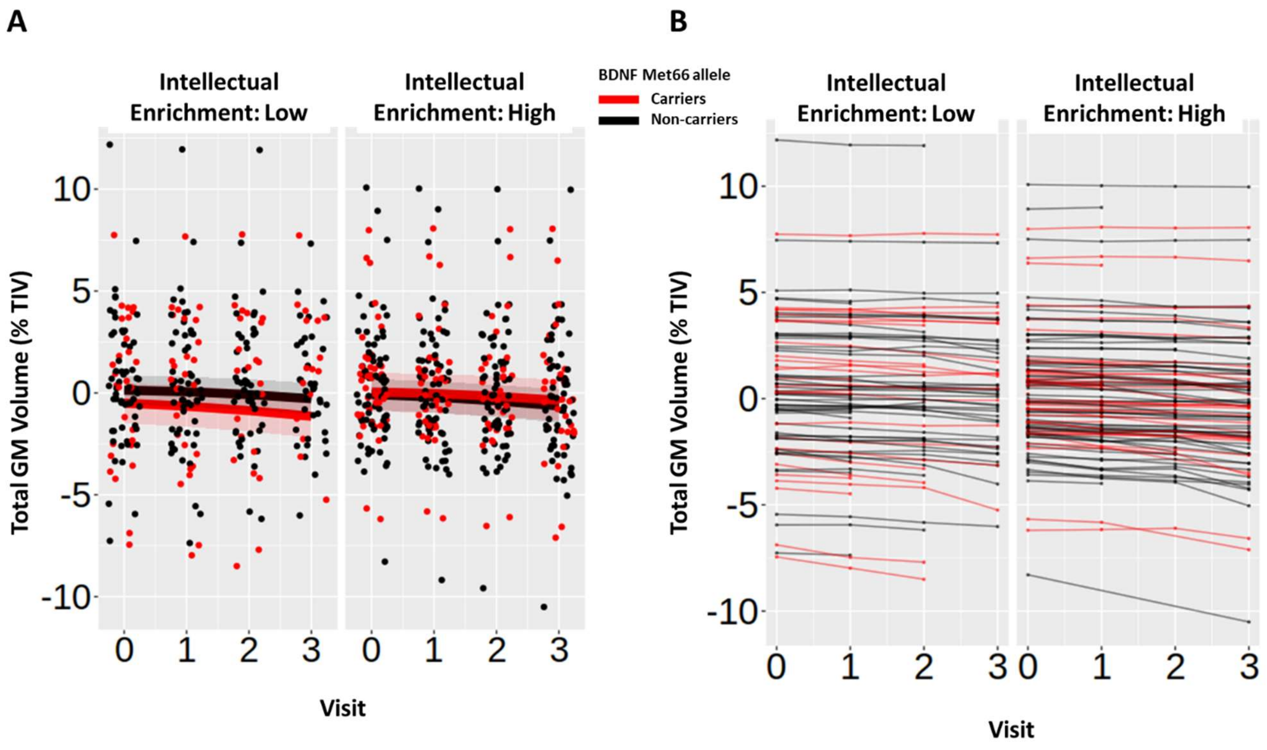

Supplementary Figure 9: Association between intellectual enrichment and *BDNF* with total GM volume as percent TIV (t-value(193.0) = 3.511, p-value < 0.001). For visualization purposes results are split into high (above mean) and low (below mean) intellectual enrichment. In (A) regression lines are generated from the mixed linear model for *BDNF* Met66 allele carriers (red) and non-carriers (black). Bands around the regression lines are 95% confidence intervals. Data have been jittered to minimize overlap. In (B) individual lines are drawn for each participant and colour coded for carriers (red) and non-carriers (black) of the Met66 allele. Datapoints in both plots show the raw data residualized against age, DBS, site and sex.
